# Supplementary material for: Dereplication of Components Coupled with HPLC-qTOF-MS in the Active Fraction of Humulus japonicus and It’s Protective Effects against Parkinson’s Disease Mouse Model
Source: Molecules. 2019 Apr 11;24(7):1435. doi: 10.3390/molecules24071435 (PMC6480934; doi:10.3390/molecules24071435)
Supplement: Supplementary file 1 [file molecules-24-01435-s001.pdf]

molecules

Supporting Contents

Dereplication of Components Coupled with HPLC-qTOF-MS in the Active Fraction of *Humulus japonicus* and it's Protective Effects Against Parkinson's Disease Mouse Model

Hee Ju Lee [1,2], Basanta Dhodary [1], Ju Yong Lee [1], Jin-Pyo An [1], Young-Kyoung Ryu [3], Kyoung-Shim Kim [3], Chul-Ho Lee [3] and Won Keun Oh [1,*]

[1] Korea Bioactive Natural Material Bank, Research Institute of Pharmaceutical Sciences, College of Pharmacy, Seoul National University, Seoul, 08826, Republic of Korea; hjlee81@kist.re.kr (H.J.L.); Basanta02@hotmail.com (B.D.); sbplee@snu.ac.kr (J.Y.L.); ntopjp77@gmail.com (J.-P.A.)
[2] Natural Product Informatics Research Center, Korea Institute of Science and Technology, Gangneung, 25451, Republic of Korea; hjlee81@kist.re.kr (H.J.L.)
[3] Laboratory Animal Resource Center, Korea Research Institute of Bioscience and Biotechnology (KRIBB), Daejeon 34141, Republic of Korea; mnc1208@kribb.re.kr (Y.-K.R.); kskim@kribb.re.kr (K.-S.K.); chullee@kribb.re.kr (C.-H.L.)
* Correspondence: wkoh1@snu.ac.kr; Tel.: +82-2-880-7872

Table of Contents

Experimental section

Fig. S1. HR-ESI-MS of compound **1**.

Fig. S2. ^{1}H NMR spectrum (CD_3OD, 500 MHz) of compound **1**.

Fig. S3. ^{13}C NMR spectrum (CD_3OD, 125 MHz) of compound **1**.

Fig. S4. HSQC spectrum (CD_3OD, 500 MHz) of compound **1**.

Fig. S5. HMBC spectrum (CD_3OD, 500 MHz) of compound **1**.

Experimental section

HPLC analysis

The HPLC system was equipped with an Agilent Series 1260 liquid chromatography, equipped with G1322A vacuum degasser, G1312C binary pump, G1329B autosampler, and G1315D DAD detector, connected to Agilent ChemStation software (Agilent Technologies Co. Ltd., Waldbronn, Germany). An INNO C18 column (4.6 x 250 mm, 5 m, Young Jin Biochrom, Korea) were used. The gradient profile was as follows: 0-3 min, initial mobile phase 0.1% formic acid in acetonitrile/0.1% formic acid in water (10:90, %, v/v); 3-43 min, linear gradient 90:10 (%, v/v); 43-52 min, isocratic 100:0 (%, v/v); and stable steps to initial condition for 8 min. Flow rate was 0.6 ml/min and detection was at 254 nm. The injection volume of standards (luteolin-7-*O*-glucoside and apigenin-7-*O*-glucoside), HJ extracts and fraction was 10 l, respectively (HPLC chromatogram data not shown).

MS Zoomed Spectrum

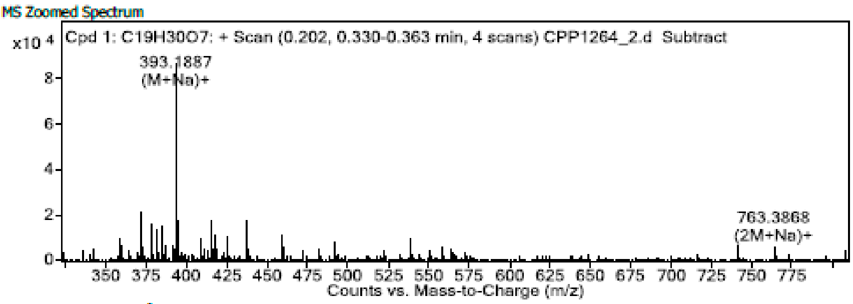

MS Spectrum Peak List

m/z	*Calc m/z*	Diff(ppm)	z	Abund	Formula	Ion
371.2068	371.2064	1.12	1	21727	C19 H31 O7	(M+H)+
375.1444	375.1778	-89.06	1	148	C19 H28 Na O6	(M+Na)+[-H2O]
391.1881	391.1517	92.87	1	1671	C19 H28 K O6	(M+K)+[-H2O]
393.1887	393.1884	0.93	1	86975	C19 H30 Na O7	(M+Na)+
409.1756	409.1623	32.36	1	9946	C19 H30 K O7	(M+K)+
723.3901	723.395	-6.86	1	461	C38 H59 O13	(2M+H)+[-H2O]
741.405	741.4056	-0.84	1	1489	C38 H61 O14	(2M+H)+
761.4044	761.3509	70.26	1	422	C38 H58 K O13	(2M+K)+[-H2O]
763.3868	763.3875	-1.01	1	6077	C38 H60 Na O14	(2M+Na)+
779.3787	779.3615	22.05	1	802	C38 H60 K O14	(2M+K)+

--- End Of Report ---

MS Zoomed Spectrum

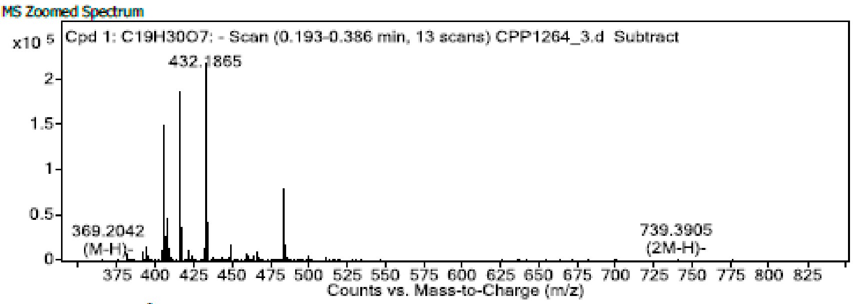

MS Spectrum Peak List

m/z	*Calc m/z*	Diff(ppm)	z	Abund	Formula	Ion
397.2115	397.1868	62.14	-1	104	C20 H29 O8	(M+HCOO)-[-H2O]
405.1674	405.1686	-2.94	-1	150172	C19 H30 Cl O7	(M+Cl)-
415.1963	415.1974	-2.64	-1	187602	C20 H31 O9	(M+HCOO)-
739.3905	739.391	-0.65	-1	1278	C38 H59 O14	(2M-H)-
757.3552	757.3571	-2.6	-1	174	C38 H58 Cl O13	(2M+Cl)-[-H2O]
767.3629	767.3859	-30.04	-1	212	C39 H59 O15	(2M+HCOO)-[-H2O]
775.3672	775.3677	-0.68	-1	934	C38 H60 Cl O14	(2M+Cl)-
785.3684	785.3965	-35.85	-1	248	C39 H61 O16	(2M+HCOO)-
801.3536	801.3066	58.57	-1	213	C38 H58 Br O13	(2M+Br)-[-H2O]
821.3391	821.316	28.15	-1	167	C38 H60 Br O14	(2M+Br)-

--- End Of Report ---

Fig. S1. HR-ESI-MS of compound **1.**

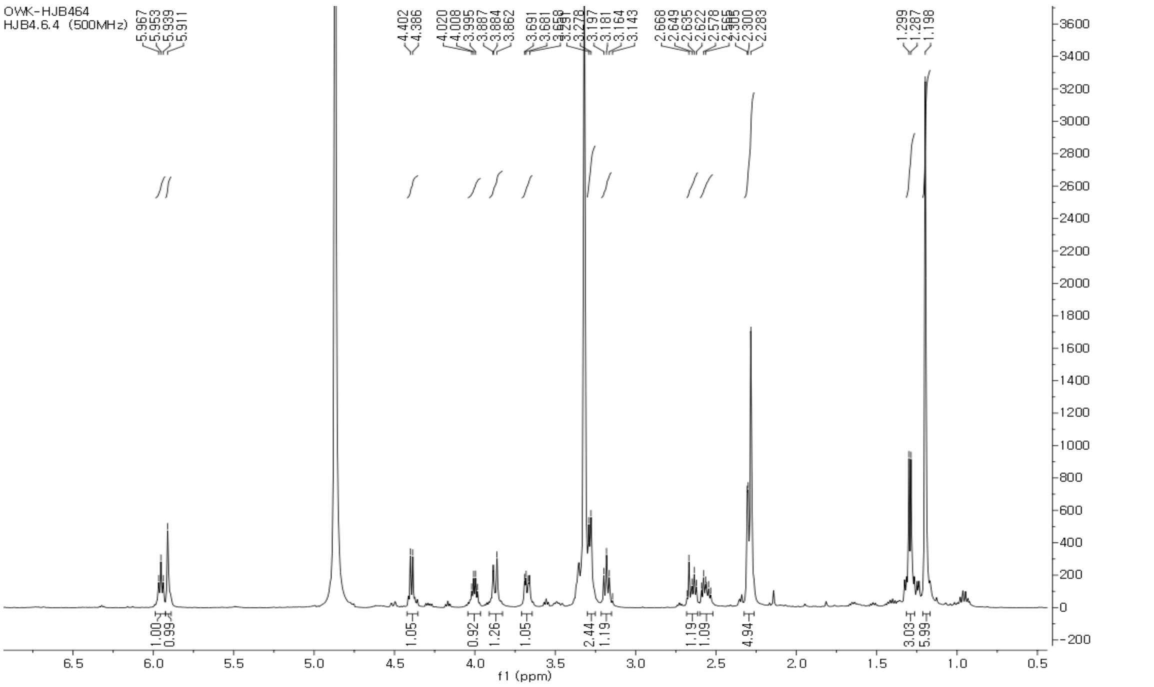

Fig. S2. ^{1}H NMR spectrum (CD_3OD, 500 MHz) of compound **1.**

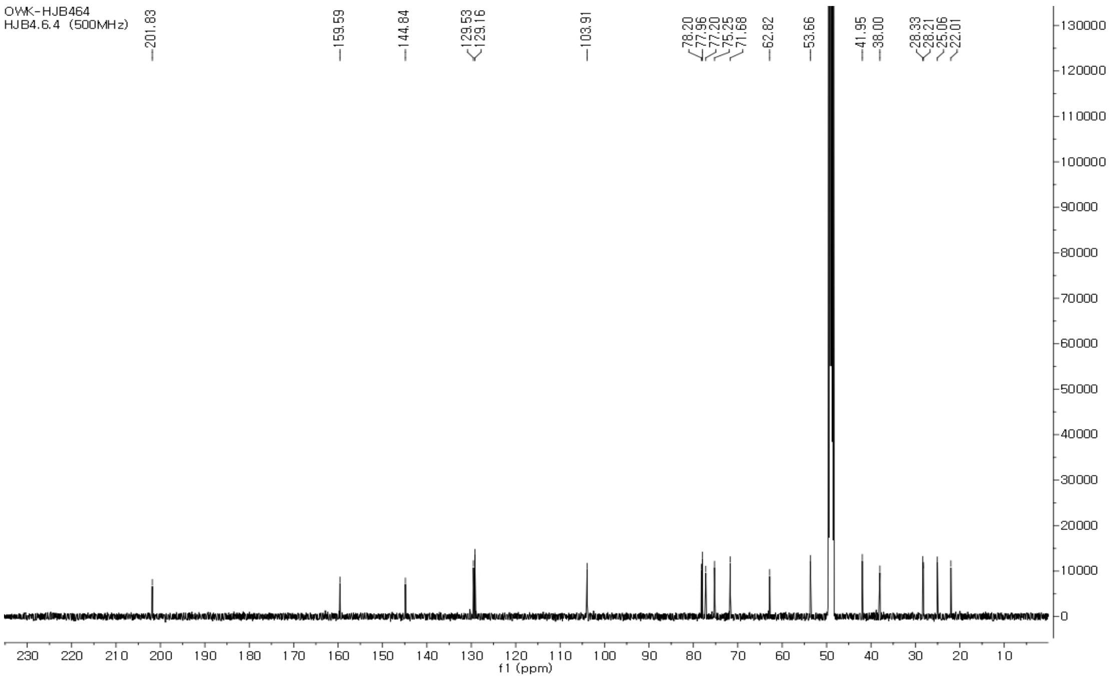

Fig. S3. ^{13}C NMR spectrum (CD_3OD, 125 MHz) of compound **1.**

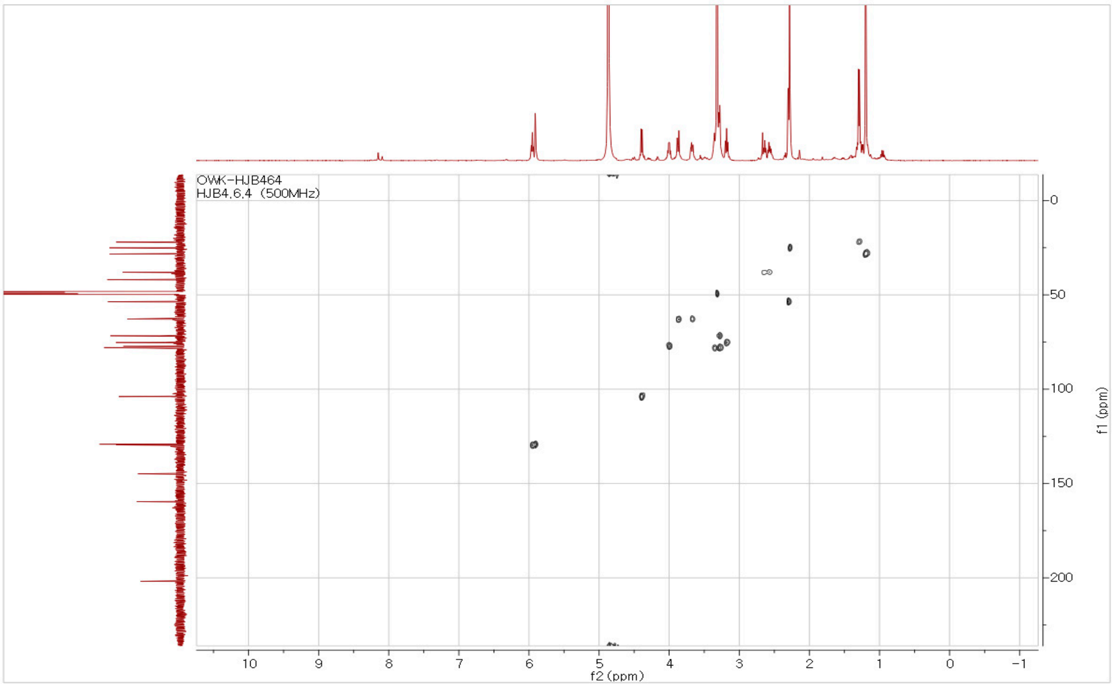

Fig. S4. HSQC spectrum (CD_3OD, 500 MHz) of compound **1.**

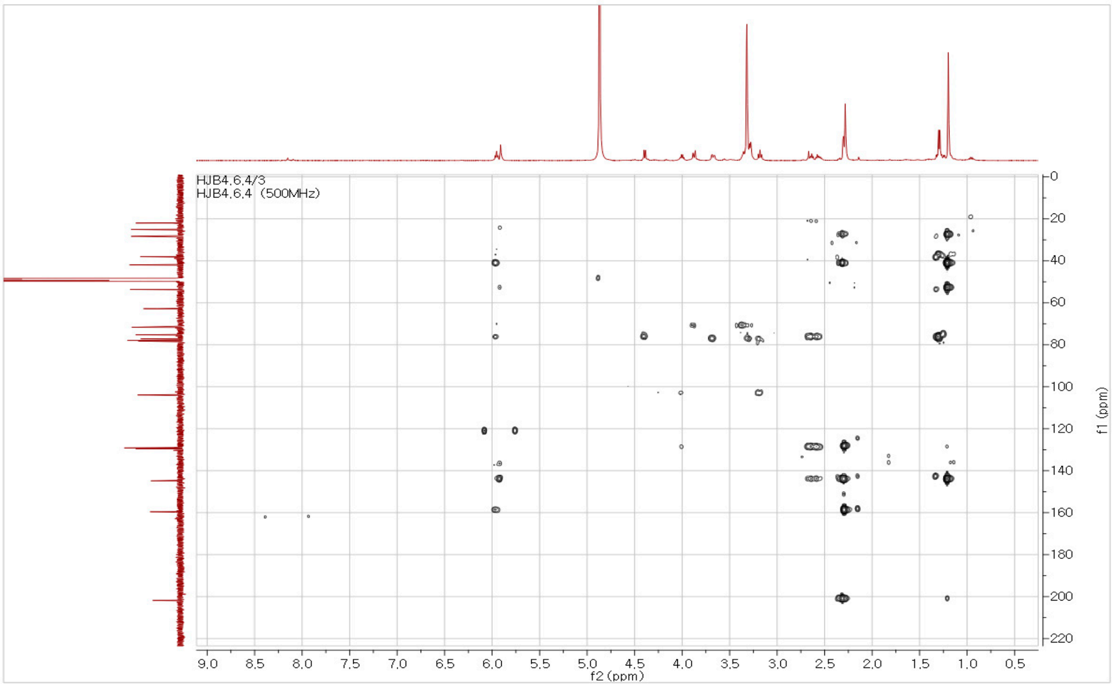

Fig. S5. HMBC spectrum (CD_3OD, 500 MHz) of compound 1.
